# 1-[(2-Chloro-7-methyl-3-quinol­yl)meth­yl]pyridin-2(1*H*)-one

**DOI:** 10.1107/S1600536810011177

**Published:** 2010-03-27

**Authors:** S. Mohana Roopan, F. Nawaz Khan, Atul Kumar Kushwaha, Venkatesha R. Hathwar, Mehmet Akkurt

**Affiliations:** a Organic and Medicinal Chemistry Research Laboratory, Organic Chemistry Division, School of Advanced Sciences, VIT University, Vellore 632 014, Tamil Nadu, India; b Solid State and Structural Chemistry Unit, Indian Institute of Science, Bangalore 560 012, Karnataka, India; c Department of Physics, Faculty of Arts and Sciences, Erciyes University, 38039 Kayseri, Turkey

## Abstract

In the title compound, C_16_H_13_ClN_2_O, the quinoline ring system is essentially planar, with a maximum deviation of 0.021 (2) Å. The pyridone ring is oriented at a dihedral angle of 85.93 (6)° with respect to the quinoline ring system. In the crystal structure, inter­molecular C—H⋯O hydrogen bonds link the mol­ecules along the *b* axis. Weak π–π stacking inter­actions [centroid–centroid distances = 3.7218 (9) and 3.6083 (9) Å] are also observed.

## Related literature

For related structures, see: Arman *et al.* (2009 ▶); Clegg & Nichol (2004 ▶); Nichol & Clegg (2005 ▶). For the synthesis of 2-pyridone derivatives, see: Conreaux *et al.* (2005 ▶); Roopan & Khan (2009 ▶); Roopan *et al.* (2010 ▶).
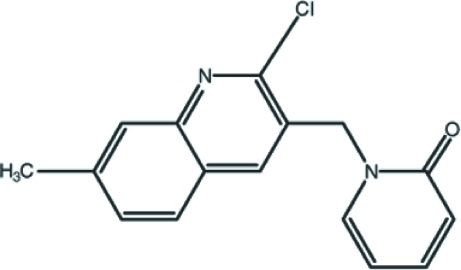


## Experimental

### 

#### Crystal data

C_16_H_13_ClN_2_O
*M* *_r_* = 284.73
Monoclinic, 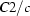

*a* = 11.8934 (3) Å
*b* = 11.1092 (3) Å
*c* = 21.2858 (6) Å
β = 102.413 (3)°
*V* = 2746.67 (13) Å^3^
*Z* = 8
Mo *K*α radiation
μ = 0.27 mm^−1^
*T* = 295 K
0.26 × 0.21 × 0.18 mm

#### Data collection

Oxford Xcalibur diffractometer with an Eos (Nova) CCD detector
Absorption correction: multi-scan (*CrysAlis PRO RED*; Oxford Diffraction, 2009 ▶) *T*_min_ = 0.932, *T*_max_ = 0.952
13638 measured reflections
2556 independent reflections
1893 reflections with *I* > 2σ(*I*)
*R*_int_ = 0.025

#### Refinement

*R*[*F* ^2^ > 2σ(*F* ^2^)] = 0.034
*wR*(*F* ^2^) = 0.095
*S* = 1.10
2556 reflections
182 parameters
H-atom parameters constrained
Δρ_max_ = 0.12 e Å^−3^
Δρ_min_ = −0.22 e Å^−3^

### 

Data collection: *CrysAlis PRO CCD* (Oxford Diffraction, 2009 ▶); cell refinement: *CrysAlis PRO CCD*; data reduction: *CrysAlis PRO RED* (Oxford Diffraction, 2009 ▶); program(s) used to solve structure: *SHELXS97* (Sheldrick, 2008 ▶); program(s) used to refine structure: *SHELXL97* (Sheldrick, 2008 ▶); molecular graphics: *ORTEP-3* (Farrugia, 1997 ▶); software used to prepare material for publication: *WinGX* (Farrugia, 1999 ▶) and *PLATON* (Spek, 2009 ▶).

## Supplementary Material

Crystal structure: contains datablocks global, I. DOI: 10.1107/S1600536810011177/is2533sup1.cif

Structure factors: contains datablocks I. DOI: 10.1107/S1600536810011177/is2533Isup2.hkl

Additional supplementary materials:  crystallographic information; 3D view; checkCIF report

## Figures and Tables

**Table 1:** Hydrogen-bond geometry (Å, °)

*D*—H⋯*A*	*D*—H	H⋯*A*	*D*⋯*A*	*D*—H⋯*A*
C13—H13⋯O1^i^	0.93	2.37	3.299 (2)	173

Symmetry code: (i) 
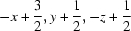
.

**Fig. 1.**
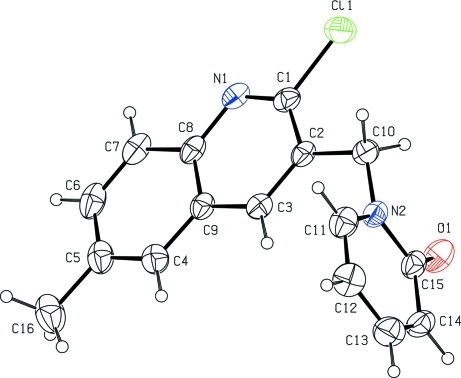
View of the title molecule with the atom numbering scheme. Displacement ellipsoids for non-H atoms are drawn at the 30% probability level.

**Fig. 2.**
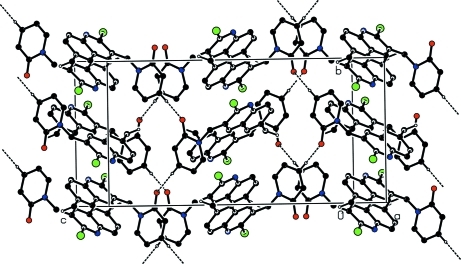
View of the packing diagram and the hydrogen bonding interactions of the title compound down the a axis. H atoms not involved in hydrogen bonding have been omitted for clarity.

## supplementary crystallographic information

### Comment

The pyridone analogues such as naturally occurring mappicine based molecule have been focused of great interest by reason of their diversified biological activities. *N*-alkylated 2-pyridones are important intermediates in the synthesis of alkaloids as illustrated by the recent synthetic approaches toward the mappicine family. Thus, modifications of biologically active mappicine synthons may lead to achieve the highly expected effective drugs (Roopan & Khan, 2009). Having succeeded in developing a practical, alternative synthesis of pyridine (Conreaux *et al.*, 2005), we then focused our attention on the general applicability of the *N*-alkylation (Roopan *et al.*, 2010) of pyridones by mean of the t-BuOK/THF system In connection with the program of synthesis of 2-pyridone analogues, we report herein the synthesis of 1-[(2-chloro-7-methylquinolin-3yl)-methyl]-pyridine-2(1*H*)-one.

In the title molecule, the quinoline ring system (N1/C1–C9) is almost planar, with maximum deviations of 0.021 (1) Å for N1 and -0.021 (2) Å for C7 (Fig. 1). The pyridone ring (N2/C11—C15) is oriented at a dihedral angle of 85.93 (6)° with respect to the quinoline ring system. In the crystal structure, intermolecular C—H···O hydrogen bonds contribute to the stability of the structure, linking the molecules along the [010] direction (Table 1 and Fig. 2). Weak π–π stacking interactions are also observed [*Cg*1···*Cg*3(3/2-*x*, 1/2-*y*, -*z*) = 3.7218 (9), where *Cg*1 and *Cg*3 are the centroids of the N1/C1–C3/C8/C9 and C4–C9 rings, respectively; *Cg*2···*Cg*2(2-*x*, *y*, 1/2-*z*) = 3.6083 (9) Å, where *Cg*2 is a centroid of the N2/C11–C15 ring].

### Experimental

To a mixed well solution of 2-pyridone (95 mg, 1 mmol, in 2 ml of DMF), KO^t^Bu (112 mg, 1 mmol, in 10 ml THF) and 2-chloro-3-(chloromethyl)-7-methylquinoline (226 mg, 1 mmol) were added and the resulting mixture was refluxed at 343 K for 1 h. After the completion of the reaction, cooled and removed the excess of solvent under reduced pressure. Crushed ice was mixed with the residue. White solid was formed, filtered, dried and purified by column chromatography using hexane and ethylacetate as the eluant. Crystals of suitable quality were grown by solvent evaporation from a diethylether solution.

### Refinement

H atoms were positioned geometrically, with C—H = 0.93, 0.96 and 0.97 Å for aromatic, methyl and methylene H, respectively, and constrained to ride on their parent atoms, with *U*_iso_(H) = 1.5*U*_eq_(C) for methyl H and *U*_iso_(H) = 1.2*U*_eq_(C) for all other H atoms.

### Crystal data

**Table:**

C_16_H_13_ClN_2_O	*F*(000) = 1184
*M**_r_* = 284.73	*D*_x_ = 1.377 Mg m^−^^3^
Monoclinic, *C*2/*c*	Mo *K*α radiation, λ = 0.71073 Å
Hall symbol: -C 2yc	Cell parameters from 985 reflections
*a* = 11.8934 (3) Å	θ = 3.4–25.5°
*b* = 11.1092 (3) Å	µ = 0.27 mm^−^^1^
*c* = 21.2858 (6) Å	*T* = 295 K
β = 102.413 (3)°	Block, colourless
*V* = 2746.67 (13) Å^3^	0.26 × 0.21 × 0.18 mm
*Z* = 8	

### Data collection

**Table:**

Oxford Xcalibur diffractometer with an Eos (Nova) CCD detector	2556 independent reflections
Radiation source: Enhance (Mo) X-ray Source	1893 reflections with *I* > 2σ(*I*)
graphite	*R*_int_ = 0.025
ω scans	θ_max_ = 25.5°, θ_min_ = 3.4°
Absorption correction: multi-scan (*CrysAlis PRO RED*; Oxford Diffraction, 2009)	*h* = −14→14
*T*_min_ = 0.932, *T*_max_ = 0.952	*k* = −13→13
13638 measured reflections	*l* = −25→25

### Refinement

**Table:**

Refinement on *F*^2^	Primary atom site location: structure-invariant direct methods
Least-squares matrix: full	Secondary atom site location: difference Fourier map
*R*[*F*^2^ > 2σ(*F*^2^)] = 0.034	Hydrogen site location: inferred from neighbouring sites
*wR*(*F*^2^) = 0.095	H-atom parameters constrained
*S* = 1.10	*w* = 1/[σ^2^(*F*_o_^2^) + (0.0497*P*)^2^ + 0.1075*P*] where *P* = (*F*_o_^2^ + 2*F*_c_^2^)/3
2556 reflections	(Δ/σ)_max_ = 0.001
182 parameters	Δρ_max_ = 0.12 e Å^−^^3^
0 restraints	Δρ_min_ = −0.22 e Å^−^^3^

### Special details

**Table:**

Geometry. Bond distances, angles etc. have been calculated using the rounded fractional coordinates. All su's are estimated from the variances of the (full) variance-covariance matrix. The cell esds are taken into account in the estimation of distances, angles and torsion angles
Refinement. Refinement on *F*^2^ for ALL reflections except those flagged by the user for potential systematic errors. Weighted *R*-factors wR and all goodnesses of fit *S* are based on *F*^2^, conventional *R*-factors *R* are based on *F*, with *F* set to zero for negative *F*^2^. The observed criterion of *F*^2^ > σ(*F*^2^) is used only for calculating -*R*-factor-obs etc. and is not relevant to the choice of reflections for refinement. *R*-factors based on *F*^2^ are statistically about twice as large as those based on *F*, and *R*-factors based on ALL data will be even larger.

### Fractional atomic coordinates and isotropic or equivalent isotropic displacement parameters (Å^2^)

**Table:**

	*x*	*y*	*z*	*U*_iso_*/*U*_eq_	
Cl1	1.09208 (3)	0.30078 (4)	0.05639 (2)	0.0632 (2)	
O1	0.85807 (10)	0.41006 (11)	0.21457 (5)	0.0685 (5)	
N1	0.89822 (11)	0.31396 (11)	−0.02677 (6)	0.0489 (5)	
N2	0.92375 (10)	0.56671 (11)	0.16368 (5)	0.0467 (4)	
C1	0.95261 (12)	0.35624 (13)	0.02798 (7)	0.0459 (5)	
C2	0.91154 (12)	0.44280 (13)	0.06637 (7)	0.0434 (5)	
C3	0.80426 (12)	0.48690 (13)	0.04162 (7)	0.0460 (5)	
C4	0.62855 (14)	0.49071 (14)	−0.04533 (8)	0.0542 (6)	
C5	0.56786 (14)	0.44843 (16)	−0.10296 (8)	0.0602 (6)	
C6	0.61864 (16)	0.35797 (17)	−0.13404 (8)	0.0648 (7)	
C7	0.72499 (15)	0.31365 (15)	−0.10943 (7)	0.0580 (6)	
C8	0.78938 (13)	0.35771 (14)	−0.05035 (7)	0.0475 (5)	
C9	0.73932 (12)	0.44607 (13)	−0.01767 (7)	0.0447 (5)	
C10	0.98427 (13)	0.48243 (15)	0.13024 (7)	0.0513 (5)	
C11	0.92446 (14)	0.68655 (15)	0.14990 (8)	0.0595 (6)	
C12	0.86613 (16)	0.76605 (17)	0.17753 (9)	0.0705 (7)	
C13	0.80463 (15)	0.72478 (18)	0.22262 (8)	0.0688 (7)	
C14	0.80364 (13)	0.60698 (17)	0.23677 (8)	0.0593 (6)	
C15	0.86079 (13)	0.51932 (16)	0.20637 (7)	0.0503 (6)	
C16	0.44951 (15)	0.49594 (19)	−0.13245 (9)	0.0845 (8)	
H3	0.77340	0.54520	0.06440	0.0550*	
H4	0.59630	0.55000	−0.02390	0.0650*	
H6	0.57750	0.32750	−0.17290	0.0780*	
H7	0.75570	0.25410	−0.13150	0.0700*	
H10A	1.00650	0.41230	0.15720	0.0620*	
H10B	1.05400	0.52020	0.12310	0.0620*	
H11	0.96660	0.71350	0.12060	0.0710*	
H12	0.86630	0.84730	0.16710	0.0850*	
H13	0.76440	0.77910	0.24280	0.0820*	
H14	0.76400	0.58190	0.26770	0.0710*	
H16A	0.43010	0.55940	−0.10600	0.1270*	
H16B	0.39420	0.43210	−0.13540	0.1270*	
H16C	0.44880	0.52660	−0.17470	0.1270*	

### Atomic displacement parameters (Å^2^)

**Table:**

	*U*^11^	*U*^22^	*U*^33^	*U*^12^	*U*^13^	*U*^23^
Cl1	0.0530 (3)	0.0666 (3)	0.0737 (3)	0.0066 (2)	0.0219 (2)	0.0046 (2)
O1	0.0847 (9)	0.0633 (8)	0.0632 (8)	−0.0139 (7)	0.0289 (6)	0.0034 (6)
N1	0.0569 (8)	0.0505 (8)	0.0446 (8)	−0.0072 (6)	0.0228 (6)	−0.0011 (6)
N2	0.0474 (7)	0.0559 (8)	0.0375 (7)	−0.0067 (6)	0.0108 (6)	−0.0030 (6)
C1	0.0485 (9)	0.0466 (9)	0.0475 (9)	−0.0040 (7)	0.0214 (7)	0.0056 (7)
C2	0.0482 (9)	0.0476 (9)	0.0372 (8)	−0.0069 (7)	0.0154 (7)	0.0024 (6)
C3	0.0492 (9)	0.0486 (9)	0.0422 (9)	−0.0028 (7)	0.0143 (7)	−0.0033 (7)
C4	0.0544 (10)	0.0542 (10)	0.0527 (10)	−0.0062 (8)	0.0085 (8)	0.0039 (8)
C5	0.0572 (10)	0.0661 (11)	0.0532 (11)	−0.0165 (9)	0.0030 (8)	0.0139 (9)
C6	0.0747 (12)	0.0765 (12)	0.0406 (10)	−0.0307 (10)	0.0064 (9)	0.0007 (9)
C7	0.0726 (12)	0.0615 (11)	0.0436 (10)	−0.0180 (9)	0.0205 (9)	−0.0052 (8)
C8	0.0578 (10)	0.0490 (9)	0.0392 (9)	−0.0138 (8)	0.0183 (7)	0.0023 (7)
C9	0.0495 (9)	0.0466 (9)	0.0394 (9)	−0.0080 (7)	0.0125 (7)	0.0033 (7)
C10	0.0462 (9)	0.0643 (10)	0.0449 (9)	−0.0019 (8)	0.0131 (7)	−0.0028 (8)
C11	0.0643 (11)	0.0594 (11)	0.0558 (11)	−0.0124 (9)	0.0150 (8)	0.0015 (8)
C12	0.0798 (13)	0.0590 (11)	0.0725 (13)	−0.0027 (10)	0.0158 (11)	−0.0053 (9)
C13	0.0641 (11)	0.0784 (14)	0.0624 (12)	0.0046 (10)	0.0105 (9)	−0.0214 (10)
C14	0.0525 (10)	0.0839 (13)	0.0437 (9)	−0.0086 (9)	0.0153 (8)	−0.0137 (9)
C15	0.0482 (9)	0.0649 (11)	0.0367 (9)	−0.0122 (8)	0.0069 (7)	−0.0054 (8)
C16	0.0655 (13)	0.0976 (16)	0.0791 (14)	−0.0176 (11)	−0.0096 (11)	0.0184 (12)

### Geometric parameters (Å, °)

**Table:**

Cl1—C1	1.7509 (15)	C11—C12	1.335 (3)
O1—C15	1.228 (2)	C12—C13	1.403 (3)
N1—C1	1.2935 (19)	C13—C14	1.344 (3)
N1—C8	1.373 (2)	C14—C15	1.421 (2)
N2—C10	1.457 (2)	C3—H3	0.9300
N2—C11	1.364 (2)	C4—H4	0.9300
N2—C15	1.3986 (19)	C6—H6	0.9300
C1—C2	1.415 (2)	C7—H7	0.9300
C2—C3	1.363 (2)	C10—H10A	0.9700
C2—C10	1.512 (2)	C10—H10B	0.9700
C3—C9	1.406 (2)	C11—H11	0.9300
C4—C5	1.366 (2)	C12—H12	0.9300
C4—C9	1.412 (2)	C13—H13	0.9300
C5—C6	1.409 (3)	C14—H14	0.9300
C5—C16	1.508 (3)	C16—H16A	0.9600
C6—C7	1.354 (3)	C16—H16B	0.9600
C7—C8	1.412 (2)	C16—H16C	0.9600
C8—C9	1.407 (2)		
			
Cl1···C9^i^	3.6496 (15)	C15···C10^iv^	3.592 (2)
Cl1···C5^ii^	3.6161 (18)	C15···C15^iv^	3.431 (2)
Cl1···C3^i^	3.5414 (15)	C16···C14^vi^	3.526 (3)
Cl1···H10A	2.8500	C5···H12^vii^	2.8400
Cl1···H10B	2.9100	C6···H14^viii^	3.0600
Cl1···H16B^ii^	3.0700	C7···H14^viii^	2.9900
O1···C2	3.3690 (18)	C8···H10B^i^	2.9900
O1···C7^ii^	3.348 (2)	C11···H3	2.7600
O1···C13^iii^	3.299 (2)	C14···H16B^vi^	2.8600
O1···H10A	2.3500	C15···H3	2.9900
O1···H13^iii^	2.3700	C16···H12^vii^	3.0100
O1···H10A^iv^	2.8600	H3···N2	2.4700
O1···H7^ii^	2.6900	H3···C11	2.7600
N2···C15^iv^	3.3835 (19)	H3···C15	2.9900
N1···H11^i^	2.8400	H3···H4	2.5000
N1···H10B^i^	2.9000	H4···H3	2.5000
N2···H3	2.4700	H4···H16A	2.3400
C1···C6^ii^	3.507 (2)	H7···O1^ii^	2.6900
C1···C7^ii^	3.550 (2)	H10A···Cl1	2.8500
C2···O1	3.3690 (18)	H10A···O1	2.3500
C2···C7^ii^	3.498 (2)	H10A···O1^iv^	2.8600
C3···C11	3.295 (2)	H10B···Cl1	2.9100
C3···C15	3.445 (2)	H10B···H11	2.3800
C3···Cl1^i^	3.5414 (15)	H10B···N1^i^	2.9000
C5···Cl1^ii^	3.6161 (18)	H10B···C8^i^	2.9900
C6···C1^ii^	3.507 (2)	H11···H10B	2.3800
C7···C2^ii^	3.498 (2)	H11···N1^i^	2.8400
C7···O1^ii^	3.348 (2)	H12···C5^vii^	2.8400
C7···C1^ii^	3.550 (2)	H12···C16^vii^	3.0100
C8···C8^ii^	3.473 (2)	H12···H16C^vii^	2.5800
C9···Cl1^i^	3.6496 (15)	H13···O1^v^	2.3700
C10···C15^iv^	3.592 (2)	H14···C6^ix^	3.0600
C11···C3	3.295 (2)	H14···C7^ix^	2.9900
C13···O1^v^	3.299 (2)	H16A···H4	2.3400
C14···C16^vi^	3.526 (3)	H16B···C14^vi^	2.8600
C15···N2^iv^	3.3835 (19)	H16B···Cl1^ii^	3.0700
C15···C3	3.445 (2)	H16C···H12^vii^	2.5800
			
C1—N1—C8	116.78 (13)	O1—C15—C14	125.67 (15)
C10—N2—C11	119.73 (12)	N2—C15—C14	114.41 (15)
C10—N2—C15	117.73 (13)	C2—C3—H3	119.00
C11—N2—C15	122.42 (13)	C9—C3—H3	119.00
Cl1—C1—N1	115.89 (11)	C5—C4—H4	119.00
Cl1—C1—C2	117.30 (11)	C9—C4—H4	119.00
N1—C1—C2	126.81 (14)	C5—C6—H6	119.00
C1—C2—C3	115.58 (13)	C7—C6—H6	119.00
C1—C2—C10	121.03 (13)	C6—C7—H7	120.00
C3—C2—C10	123.39 (13)	C8—C7—H7	120.00
C2—C3—C9	121.28 (14)	N2—C10—H10A	109.00
C5—C4—C9	121.27 (15)	N2—C10—H10B	109.00
C4—C5—C6	118.03 (16)	C2—C10—H10A	109.00
C4—C5—C16	121.28 (16)	C2—C10—H10B	109.00
C6—C5—C16	120.68 (16)	H10A—C10—H10B	108.00
C5—C6—C7	122.49 (16)	N2—C11—H11	119.00
C6—C7—C8	120.10 (15)	C12—C11—H11	119.00
N1—C8—C7	119.42 (14)	C11—C12—H12	121.00
N1—C8—C9	122.15 (13)	C13—C12—H12	121.00
C7—C8—C9	118.43 (14)	C12—C13—H13	120.00
C3—C9—C4	122.97 (14)	C14—C13—H13	120.00
C3—C9—C8	117.37 (13)	C13—C14—H14	119.00
C4—C9—C8	119.65 (14)	C15—C14—H14	119.00
N2—C10—C2	112.30 (12)	C5—C16—H16A	109.00
N2—C11—C12	121.55 (16)	C5—C16—H16B	109.00
C11—C12—C13	118.81 (17)	C5—C16—H16C	109.00
C12—C13—C14	120.18 (17)	H16A—C16—H16B	109.00
C13—C14—C15	122.51 (16)	H16A—C16—H16C	109.00
O1—C15—N2	119.91 (14)	H16B—C16—H16C	109.00
			
C8—N1—C1—Cl1	−179.03 (11)	C2—C3—C9—C4	−179.11 (15)
C8—N1—C1—C2	0.2 (2)	C2—C3—C9—C8	0.1 (2)
C1—N1—C8—C7	−179.03 (14)	C9—C4—C5—C6	0.5 (2)
C1—N1—C8—C9	1.3 (2)	C9—C4—C5—C16	179.90 (16)
C11—N2—C10—C2	−85.11 (17)	C5—C4—C9—C3	−179.74 (15)
C15—N2—C10—C2	91.08 (15)	C5—C4—C9—C8	1.0 (2)
C10—N2—C11—C12	177.19 (16)	C4—C5—C6—C7	−1.1 (3)
C15—N2—C11—C12	1.2 (2)	C16—C5—C6—C7	179.45 (17)
C10—N2—C15—O1	0.1 (2)	C5—C6—C7—C8	0.2 (3)
C10—N2—C15—C14	−179.61 (13)	C6—C7—C8—N1	−178.43 (15)
C11—N2—C15—O1	176.14 (14)	C6—C7—C8—C9	1.3 (2)
C11—N2—C15—C14	−3.5 (2)	N1—C8—C9—C3	−1.5 (2)
Cl1—C1—C2—C3	177.80 (11)	N1—C8—C9—C4	177.83 (14)
Cl1—C1—C2—C10	−1.85 (19)	C7—C8—C9—C3	178.85 (14)
N1—C1—C2—C3	−1.4 (2)	C7—C8—C9—C4	−1.9 (2)
N1—C1—C2—C10	178.91 (14)	N2—C11—C12—C13	1.2 (3)
C1—C2—C3—C9	1.2 (2)	C11—C12—C13—C14	−0.9 (3)
C10—C2—C3—C9	−179.19 (14)	C12—C13—C14—C15	−1.7 (3)
C1—C2—C10—N2	−176.75 (13)	C13—C14—C15—O1	−175.85 (16)
C3—C2—C10—N2	3.6 (2)	C13—C14—C15—N2	3.8 (2)

Symmetry codes: (i) −*x*+2, −*y*+1, −*z*; (ii) −*x*+3/2, −*y*+1/2, −*z*; (iii) −*x*+3/2, *y*−1/2, −*z*+1/2; (iv) −*x*+2, *y*, −*z*+1/2; (v) −*x*+3/2, *y*+1/2, −*z*+1/2; (vi) −*x*+1, −*y*+1, −*z*; (vii) −*x*+3/2, −*y*+3/2, −*z*; (viii) *x*, −*y*+1, *z*−1/2; (ix) *x*, −*y*+1, *z*+1/2.

### Hydrogen-bond geometry (Å, °)

**Table:**

*D*—H···*A*	*D*—H	H···*A*	*D*···*A*	*D*—H···*A*
C13—H13···O1^v^	0.93	2.37	3.299 (2)	173

Symmetry codes: (v) −*x*+3/2, *y*+1/2, −*z*+1/2.
